# Gender Differences in Risks of Coronary Heart Disease and Stroke in Patients with Type 2 Diabetes Mellitus and Their Association with Metabolic Syndrome in China

**DOI:** 10.1155/2016/8483405

**Published:** 2016-11-30

**Authors:** Mei-Fang Yao, Jie He, Xue Sun, Xiao-Li Ji, Yue Ding, Yi-Ming Zhao, Han-Yu Lou, Xiao-Xiao Song, Li-Zhen Shan, Ying-Xiu Kang, Song-Zhao Zhang, Peng-Fei Shan

**Affiliations:** ^1^Department of Endocrinology and Metabolism, The Second Affiliated Hospital, Zhejiang University College of Medicine, Hangzhou, Zhejiang, China; ^2^Health Management Center, Zhejiang Hospital, Hangzhou 310013, China; ^3^Department of Clinical Laboratory, The Second Affiliated Hospital, Zhejiang University College of Medicine, Hangzhou, Zhejiang, China

## Abstract

Coronary heart disease (CHD) and stroke are common complications of type 2 diabetes mellitus (T2DM). We aimed to explore the differences in the risks of CHD and stroke between Chinese women and men with T2DM and their association with metabolic syndrome (MS). This study included 1514 patients with T2DM. The Asian Guidelines of ATPIII (2005) were used for MS diagnosis, and the UKPDS risk engine was used to evaluate the 10-year CHD and stroke risks. Women had lower CHD risk (15.3% versus 26.3%), fatal CHD risk (11.8% versus 19.0%), stroke risk (8.4% versus 10.3%), and fatal stroke risk (1.4% versus 1.6%) compared with men with T2DM (*p* < 0.05–0.001). The CHD risk (28.4% versus 22.6%, *p* < 0.001) was significantly higher in men with MS than in those without MS. The CHD (16.2% versus 11.0%, *p* < 0.001) and stroke risks (8.9% versus 5.8%, *p* < 0.001) were higher in women with MS than in those without MS. In conclusion, our findings indicated that Chinese women with T2DM are less susceptible to CHD and stroke than men. Further, MS increases the risk of both these events, highlighting the need for comprehensive metabolic control in T2DM.

## 1. Introduction

With lifestyle changes and increased longevity, cardiovascular (CVD) and cerebrovascular diseases have become a common threat to human health and are the leading cause of death in many countries [1]. These conditions are also common macrovascular complications of type 2 diabetes mellitus (T2DM) and the main cause of T2DM-related mortality [2]. Compared to nondiabetic individuals, patients with T2DM have a 1.54–4 times higher risk of coronary heart disease (CHD) [3], along with an earlier age of onset, greater lesion severity, and a poorer prognosis. Studies have also shown that the morbidity of cerebrovascular disease is 2–5 times higher in patients with T2DM than in nondiabetic individuals [4]. In the general population, women usually have a 10-year delay in the onset of subclinical atherosclerosis [5] and cardiovascular events compared to men. However, some studies in Western countries have demonstrated that women with diabetes seem less likely to achieve therapeutic goals for blood pressure, low-density lipoprotein (LDL) cholesterol (LDL-c), and glycosylated hemoglobin (HbA1c) [6] and even have a higher risk for CHD than diabetic men. Lifestyle factors like smoking and alcohol consumption are also well known to increase the risk of CVD and cerebrovascular disease. Chinese women smoke less than women in the West [7], and they also have lower prevalence of alcohol dependence [8]. However, metabolic control in Chinese women with T2DM has not been clearly elucidated, and it remains largely unknown whether the risk of CVD and cerebrovascular disease in women with T2DM is higher than that in men with T2DM.

Metabolic syndrome (MS) is a cluster of clinical syndromes characterized by multiple principal diseases or risk factors such as abnormal glucose metabolism (diabetes or impaired glucose regulation), high blood pressure, dyslipidemia, and central obesity [5]. Although in the general population MS increases the risk of CVD and cerebrovascular disease [9], whether MS further increases the risk of CVD and cerebrovascular disease in patients with T2DM has not been definitively determined: several studies suggest that MS does not increase the risk [10, 11], while others have shown that it does [12–14]. To our knowledge, research on this matter, especially among Chinese patients, is rather limited.

In the present study, we adopted the UK Prospective Diabetes Study (UKPDS) risk engine to predict the risk of CHD and stroke in the next 10 years [15], compared the CVD risk factors and 10-year risks of CHD and stroke between Chinese women and men with T2DM, and analyzed the association between MS and 10-year risks of CHD and stroke in T2DM. The study aimed to explore gender differences in the risk of CHD and stroke in T2DM and investigate whether the risk of CVD and cerebrovascular disease is greater in T2DM complicated with MS than in T2DM alone.

## 2. Subjects and Methods

### 2.1. Subjects

Patients with T2DM were enrolled from those hospitalized in the Second Affiliated Hospital, Zhejiang University School of Medicine, from May 2008 to April 2013. All patients met the 2006 World Health Organization criteria for the diagnosis and classification of diabetes. Those with type 1 diabetes mellitus, other types of diabetes mellitus, gestational diabetes, GAD antibody positivity, severe edema, and ascites as determined by abdominal ultrasound and those taking corticosteroids were excluded. Further, 72 patients with CHD, 68 patients with stroke, and 5 patients with CHD and stroke were additionally excluded, leaving a total of 1514 cases (796 men and 718 women) that were included in the statistical analysis. The ages of these patients ranged from 30 to 79 years, and the duration of diabetes varied from 3 days to 36 years. This study was approved by the Ethics Committee of the Second Affiliated Hospital, Zhejiang University School of Medicine, and all subjects provided informed consent for participation.

### 2.2. Methods

The medical history of all patients was recorded, including the duration of diabetes, smoking history, hypertension history, and history of taking antihypertensive and hypoglycemic drugs. Duration of diabetes was defined as years since diagnosis of diabetes which was reported by patients or by at least one first-degree relative of patients and confirmed by record of medical history. “Current smokers” were defined as those who smoked during the period of the study. “Former smokers” were defined as those who used to smoke but had quit for more than 12 months before the start of the study. All subjects underwent a complete physical examination, including weight, height, and blood pressure. A calibrated standard balance-beam scale and standard height bar were used to measure weight and height, respectively. Body mass index (BMI) was calculated as weight (kg)/(the square of height) (m^2^). Blood pressure was measured twice using an automatic blood pressure monitor on the right upper arm with the participant seated/or in the decubitus position. The average of the two measurements was used as the blood pressure in the analysis.

Venous blood was collected between 6:00 am and 9:00 am after an overnight (8–12 hours) fast. A TOSOH HLC-723G8 automatic glycohemoglobin analyzer (Tosoh Corporation, Yamaguchi 746-0042, Japan) was used to assay HbA1c, and an Olympus AU4500 automatic chemistry analyzer (Olympus Corporation, Tokyo, Japan) was used to determine the levels of fasting blood glucose (FBG), total cholesterol (TC), triglycerides (TG), LDL-c, and high-density lipoprotein cholesterol (HDL-c).

### 2.3. Diagnostic Criteria for MS

We adopted the Asian Guidelines of National Cholesterol Education Program Adult Treatment Panel III (ATPIII) (2005) for MS diagnosis [16], according to which at least three of the following conditions had to be met: (1) abdominal obesity [waist circumference (WC) ≥ 90 cm (men) or ≥ 80 cm (women)]; (2) high blood TG levels (>1.7 mmol/L or receiving treatment for this); (3) reduced blood HDL-c levels [<1.04 mmol/L (men) or < 1.29 mmol/L (women)]; (4) systolic blood pressure (SBP)/diastolic blood pressure (DBP) ≥ 130/85 mmHg, receiving hypertensive treatment, or a previous diagnosis of hypertension; (5) high blood glucose levels (FPG ≥ 5.6 mmol/L, receiving hyperglycemia treatment, or a previous diagnosis of T2DM).

### 2.4. CHD and Stroke Risk Assessment

We used the UKPDS risk engine [15] to calculate the 10-year CHD risk, fatal CHD risk, stroke risk, and fatal stroke risk on the basis of current age; duration of diabetes; levels of HbA1c, TC, and HDL-c; SBP; and presence of atrial fibrillation (AF); smoking status; gender; and race. All patients were divided into low-risk, moderate-risk, and high-risk groups depending on the CHD risk (>20% for high risk, 10%–20% for moderate risk, and <10% for low risk).

### 2.5. Statistical Analysis

SPSS 20.0 statistical software was used for data processing. Measurement data were expressed as means ± standard deviation (SD) or median (25th–75th percentiles), and categorical variables were reported as percentages. The independent samples' *t*-test or 2 independent samples' nonparametric test was used for comparison between groups, and ANOVA was used for comparison between three or more groups. Patients were grouped according to gender, the presence or absence of MS, and age (groups of 10 years), and the CHD risk, fatal CHD risk, stroke risk, and fatal stroke risk were calculated for each age group. Patients were also grouped according to the components of MS among women and men, and the differences in CVD and stroke risk were compared among the component groups by using a nonparametric test (Kruskal-Wallis test). The odds ratio (OR) of high CHD risk in women and men with different MS components was determined using binary logistic regression analysis, with a high 10-year CHD risk (>20%) as the dependent variable and the components of MS as the independent variables. A *p* value less than 0.05 was considered statistically significant.

## 3. Results

### 3.1. Comparison of Clinical and Biochemical Data between Women and Men with T2DM

The age and years since diagnosis of diabetes were greater in women than in men, as was the lipid profile (*p* < 0.01–0.001 for all) (Table 1). In contrast, the WC, DBP, and HbA1c levels, current smoking rate, and former smoking rates were significantly lower in women than in men (*p* < 0.05–0.001) (Table 1). No significant differences were found in BMI and FBG levels between women and men with T2DM (Table 1).

### 3.2. Comparison of CHD and Stroke Risk between Women and Men with T2DM

The CHD risk (15.3%  ± 10.7% versus 26.3%  ± 17.2%, *p* < 0.001), fatal CHD risk (11.8%  ± 10.0% versus 19.0%  ± 15.9%, *p* < 0.001), stroke risk (8.4%  ± 10.6% versus 10.3%  ± 12.1%, *p* < 0.005), and fatal stroke risk (1.4%  ± 2.0% versus 1.6%  ± 2.3%, *p* < 0.05) were all significantly lower in women than in men with T2DM.

### 3.3. Comparison of CHD and Stroke Risk between Patients with T2DM Who Had MS and Those without MS

**Figure 1:**
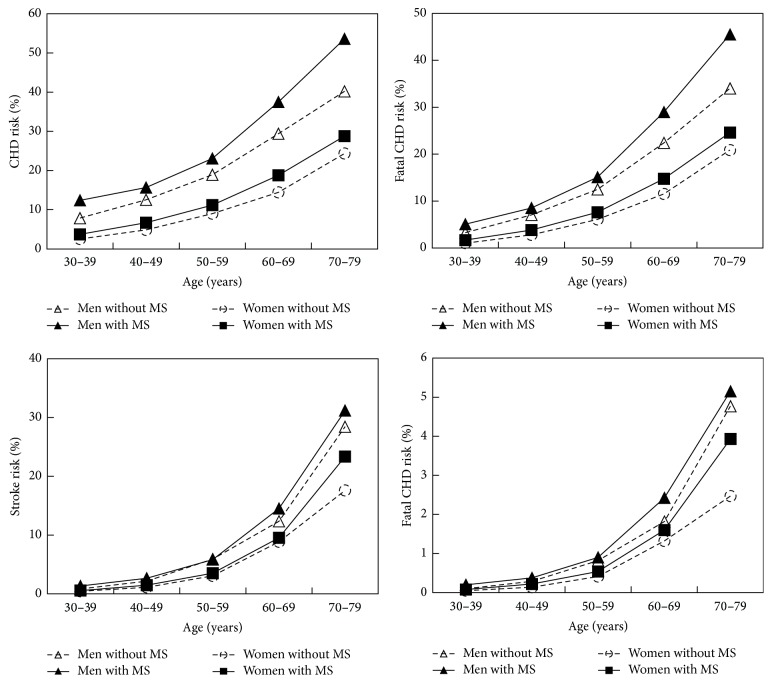
Age-related changes in CHD, fatal CHD, stroke, and fatal stroke risks according to MS status and gender. CHD: coronary heart disease; MS: metabolic syndrome.

Compared with women with T2DM but without MS, women with T2DM and MS had higher CHD risk, fatal CHD risk, stroke risk, and fatal stroke risk (*p* < 0.001 for all) (Table 2). Compared with men with T2DM but without MS, men with T2DM and MS had higher CHD risk and fatal CHD risk (*p* < 0.001 for both); similarly, the stroke risk and fatal stroke risk tended to be higher in men with T2DM and MS, although this difference was not significant (Table 2). All four types of risk increased with age and all were higher in the MS group than in the non-MS group, irrespective of the age group (Figure 1).

Compared with men with MS, women with MS had 12.2%, 8.1%, and 1.7% lower risks of CHD, fatal CHD, and stroke (*p* < 0.05–0.001), respectively. Compared with men without MS, women without MS had 11.6%, 8.1%, 4.0%, and 0.7% lower risks in CHD, fatal CHD, stroke, and fatal stroke (*p* < 0.001), respectively.

### 3.4. Effect of Number of MS Components on the UKPDS Risk Score

The CHD risk, fatal CHD risk, stroke risk, and fatal stroke risk gradually increased with the increase in the number of MS components in patients with T2DM (*p* = 0.017–0.000) (Table 3). Binary logistic regression analysis showed that, with the increase in the number of MS components, the proportion of patients with a high 10-year CHD risk also increased, with a corresponding increase in the OR (*p* < 0.05–0.001) (Table 4).

## 4. Discussion

The results of the present large-scale study showed that the TC and LDL-c levels were higher in women with T2DM than in men with T2DM, a finding consistent with those of previous studies [6, 17]. By studying patients with T2DM from 236 diabetes centers in Italy, Russo et al. found that, compared to men, women had higher levels of TC, HDL-c, and LDL-c, were older, and had diabetes for a longer duration; further, more women than men failed to achieve the control goal of LDL-c levels [17]. Similarly, the eControl Study showed that, in the overall Spanish population, although the proportion of women on lipid-lowering therapy was slightly higher than that of men, the LDL-c levels in women were significantly higher than those in men; thus, irrespective of whether they have CVD as a complication, it is more difficult for women with T2DM to achieve the control goals of BMI and LDL-c level than it is for men [6]. The differences in the TC and LDL-c levels between genders may be related to differences in the pattern of changes of TC and LDL-c levels with age. In women, TC and LDL-c levels increase with age, especially after menopause, because of the lowered estrogen level; in contrast, blood lipid levels peak in men around the age of 40.

Another important result of this study was that DBP and HbA1c levels were significantly lower in women with T2DM than in men with T2DM, although no significant differences were found in BMI and SBP. This may be the main reason for the significantly lower CHD and stroke risks in Chinese women with T2DM than in men with T2DM. Gender differences are known to exist in CVD risk factor control in various countries and races. Like the eControl Study conducted in Spain [6], Collier et al. found that BMI, TC and HDL-c levels, and the proportion of hypertension were all higher in Scottish women with T2DM than in men. Further, although no differences were found in HbA1c levels, the risks of peripheral vascular disease, ischemic heart disease, and stroke were significantly lower in women than in men [18]. The SBP and LDL-c levels were significantly higher in women with T2DM from South Carolina than in men, although no significant differences were found in HbA1c levels and DBP [19]. Compared to men, Italian women with T2DM were older and had higher levels of HbA1c, TG, TC, LDL-c, and HDL-c and higher BMI and SBP [20]. Schroeder et al. found that the LDL-c levels and SBP were higher in American women newly diagnosed with T2DM than in men, while HbA1c levels and DBP were lower; after treatment for one year, the differences in HbA1c levels between the genders disappeared and the differences in blood pressure and LDL-c levels narrowed [21].

The rate of current smoking was 1.1% among the women in this study, which was significantly lower than that among the men. The smoking rate among women was also slightly lower than that in the general population (46.71% for Chinese men and 5.34% for Chinese women) [7]. Recently, the morbidity and mortality of CVD and cerebrovascular diseases have decreased in America [22] as a result of good control of CVD risk factors, of which decreased smoking rate is an important one. In the Study of Inherited Risk of Coronary Atherosclerosis and the Penn Diabetes Heart Study, the current smoker rate was as low as 11.5% among men with T2DM and 10.1% among women [23]. Thus, the low smoking rate among women with T2DM in our study may be another important reason for the lower morbidity of CVD and cerebrovascular diseases in this group than in men.

In a previous study, we showed that, in the general population, MS increased the risks of carotid atherosclerosis and carotid plaques, which increased further with the number of MS components [5]. In the present study as well, the risks of CHD and stroke were higher in the MS group than in the non-MS group and increased gradually with the number of MS components, along with the proportion of patients with high CHD risk. Additionally, weight, BMI, WC, blood pressure, and levels of TC, TG, and LDL-c were higher in the MS group than in the non-MS group, while the HDL-c level showed the opposite trend.

Recent studies have shown that both MS and T2DM significantly increase the risk of CVD and cerebrovascular diseases [24, 25], but few studies have examined the association between MS and the risk of CVD and cerebrovascular diseases in patients with T2DM, and their results have been conflicting. The cross-sectional study conducted by Rhee et al. found that MS increased the risk of CVD events in the normal population and population with abnormal glucose tolerance but did not increase the CVD risk in the diabetic population [10]. Bae et al.'s study [11] with an average follow-up period of 8.0 years and 8898 cases found that both diabetes and MS significantly increased the occurrence of cardiovascular events and the probability of CVD-related mortality, but, in patients with diabetes, MS did not significantly increase either. On the other hand, through their cross-sectional study, Sone et al. found that MS was an independent risk factor for coronary atherosclerosis [13]. Sone et al. conducted a prospective study for 8 years on 1424 patients with T2DM in Japan and found that MS was a predictor of CVD but with limited prediction value, and the predictive function of the MS components hyperlipidemia and hypertension was equivalent to or better than that of MS [13]. In the Fenofibrate Intervention and Event Lowering in Diabetes trial, Scott et al. analyzed 4900 diabetic patients in the placebo group and found that the numbers of cardiovascular events were higher in the population with diabetes and MS than that in the population with diabetes alone. Further, they found that MS was a CVD risk factor independent of traditional risk factors, low HDL-c levels combined with hypertriglyceridemia increased the CVD risk by 41%, and each 10 mmHg increase in blood pressure for patients without CVD at baseline increased the CVD risk by 16% [14]. The reasons for the inconsistencies in research findings are not yet clear, but one possible reason could be the fact that since the diabetic population with MS may have a combination of a large number of CVD risk factors, these patients receive more comprehensive blood sugar-, blood pressure-, and blood lipid-lowering treatment, which in turn reduces CHD-related morbidity and mortality [22] and attenuates the effects of MS on the risk of CHD and stroke in T2DM.

## 5. Conclusion

The present large-scale study on Chinese patients with T2DM showed that the 10-year risks of CHD and stroke were lower in women than in men with T2DM. The risks of CHD and stroke were also lower in women with MS than in men with MS. In addition, MS complicating T2DM increased the 10-year risk of CVD and cerebrovascular disease in both men and women with T2DM. The 10-year risk of CHD and stroke also increased with the number of MS components. Considering that cardiovascular complications are an important cause of death in patients with T2DM, comprehensive treatment, including therapy for lowering blood glucose levels, blood pressure, and blood lipid levels, would be beneficial to reduce diabetes complications, the occurrence of cardiovascular and cerebrovascular events, and the related mortality.

## Figures and Tables

**Table 1:** Anthropometric and biochemical data of subjects according to gender.

	Men	Women
*n*	796	718
Age (years)	56.2 ± 11.6	59.4 ± 10.4^‡^
YSDD (years)	7.1 ± 6.3	8.6 ± 6.7^‡^
Weight (Kg)	69.4 ± 11.3	59.0 ± 10.4^‡^
BMI (Kg/m^2^)	24.1 ± 3.4	23.8 ± 3.7
WC (cm)	88.8 ± 9.8	86.5 ± 10.3^‡^
Systolic BP (mmHg)	136.6 ± 20.1	137.9 ± 20.4
Diastolic BP (mmHg)	82.8 ± 11.4	81.6 ± 10.7^*∗*^
FBG (mmol/L)	9.5 ± 4.0	9.1 ± 3.7
HbA1c (%)	9.8 ± 2.5	9.5 ± 2.4^*∗*^
TC (mmol/L)	4.4 (3.8, 5.2)	4.7 (4.0, 5.5)^‡^
Triglyceride (mmol/L)	1.5 (1.1, 2.1)	1.7 (1.2, 2.3)^†^
HDL-c (mmol/L)	1.1 (1.0, 1.4)	1.3 (1.1, 1.5)^‡^
LDL-c (mmol/L)	2.8 (2.2, 3.5)	2.9 (2.4, 3.5)^†^
Current smoker (*n*, %)	326 (41.0%)	8 (1.1%)^‡^
Former smoker (*n*, %)	172 (21.6%)	3 (0.4%)^‡^
AF (*n*, %)	6 (0.8%)	6 (0.8%)

^*∗*^ *p* < 0.05, ^†^ *p* < 0.01, and ^‡^ *p* < 0.001 compared to men. Data are expressed as means ± standard deviation (SD) or median (25th–75th percentiles), and categorical variables were reported as percentages. WC: waist circumference; BP: blood pressure; FBG: fasting blood glucose; TC: total cholesterol; HDL-c: high-density lipoprotein cholesterol; LDL-c: low-density lipoprotein cholesterol; AF: atrial fibrillation; YSDD: years since diagnosis of diabetes.

**Table 2:** CHD risk and stroke risk in subjects depending on gender and the presence or absence of metabolic syndrome.

	Men		Women	
	Non-MS	MS	Non-MS	MS
CHD risk (%)	22.6 ± 14.4	28.4 ± 18.3^‡^	11.0 ± 8.4^#^	16.2 ± 10.9^‡†^
Fatal CHD risk (%)	16.4 ± 13.3	20.6 ± 17.0^‡^	8.3 ± 7.7^#^	12.5 ± 10.3^‡†^
Stroke risk (%)	9.8 ± 12.0	10.6 ± 12.2	5.8 ± 6.2^#^	8.9 ± 11.3^‡*∗*^
Fatal stroke risk (%)	1.5 ± 2.4	1.7 ± 2.2	0.8 ± 0.9^#^	1.5 ± 2.2^‡^

^‡^ *p* < 0.001 compared to subjects of the same gender without MS. ^#^ *p* < 0.001 compared to men without MS. ^*∗*^ *p* < 0.05 and ^†^ *p* < 0.001 compared to men with MS. Data are expressed as mean ± standard deviation. CHD: coronary heart disease; MS: metabolic syndrome.

**Table 3:** CHD risk and stroke risk in subjects depending on the number of MS components.

		Men			Women	
	*n*	CHD risk (%)	Stroke risk (%)	*n*	CHD risk (%)	Stroke risk (%)
1	62	17.5 ± 11.4	5.0 ± 5.55	23	6.3 ± 5.0	3.1 ± 5.1
2	231	24.0 ± 14.9	11.1 ± 13.0	102	12.1 ± 8.6	6.4 ± 6.3
3	235	26.6 ± 17.0	10.6 ± 12.0	191	13.6 ± 9.2	8.5 ± 12.5
4	182	29.8 ± 18.6	11.5 ± 13.1	236	16.8 ± 11.5	9.5 ± 12.3
5	86	30.6 ± 20.7	8.7 ± 10.6	166	18.4 ± 11.3	8.6 ± 7.8
*F* value		8.65	4.14		13.3	3.0
*p*		0.000	0.003		0.000	0.017

Data are expressed as mean ± SD. CHD: coronary heart disease; MS: metabolic syndrome.

**Table 4:** Gender-specific presence and OR of high CHD risk depending on the number of MS components.

	*n*	CHD, *n* (%)	OR (95% CI)	Stroke, *n* (%)	OR (95% CI)
Men					
1	62	16 (25.8%)	1.0	2 (3.2%)	1.0
2	231	121 (52.4%)	3.16 (1.69, 5.91)^‡^	33 (14.3%)	5.0 (1.2, 21.4)^*∗*^
3	235	126 (53.6%)	3.32 (1.78, 6.20)^‡^	43 (18.2%)	6.7 (1.6, 28.6)^*∗*^
4	182	114 (62.6%)	4.82 (2.53, 9.17)^‡^	27 (14.8%)	5.2 (1.2, 22.7)^*∗*^
5	86	51 (59.3%)	4.19 (2.05, 8.55)^‡^	10 (19.6%)	3.9 (0.833, 18.7)
Women					
1	23	1 (4.3%)	1.0	1 (4.3%)	1.0
2	102	17 (16.7%)	4.40 (0.56, 34.9)	4 (3.9%)	0.9 (0.1, 8.4)
3	191	41 (21.5%)	6.01 (0.79, 45.9)	18 (9.4%)	2.3 (0.3, 18.0)
4	236	74 (31.4%)	10.0 (1.33, 76.0)^*∗*^	27 (11.3%)	2.8 (0.4, 21.9)
5	166	58 (34.9%)	11.8 (1.55, 89.9)^*∗*^	12 (7.2%)	1.7 (0.2, 13.8)

^*∗*^ *p* < 0.05 and ^‡^ *p* < 0.001 compared to subjects with type 2 diabetes mellitus without other components of MS. The 10-year CHD and stroke risk were estimated using the UKPDS risk engine with individuals categorized as high risk (>20% risk). CHD: coronary heart disease; MS: metabolic syndrome; OR: odds ratio.
